# From graft survival to lived experience: a new research agenda in transplant medicine

**DOI:** 10.3389/ti.2026.17264

**Published:** 2026-10-05

**Authors:** Chiara Becchetti, Sofia C. Zambrano

**Affiliations:** 1 Graduate School for Health Sciences (GHS), University of Bern, Bern, Switzerland; 2 Department of Visceral Surgery and Medicine, Inselspital, Bern University Hospital, University of Bern, Bern, Switzerland; 3 Hepatology and Gastroenterology Division, ASST Grande Ospedale Metropolitano Niguarda, Niguarda Hospital, Milan, Italy; 4 Institute of Social and Preventive Medicine, ISPM, University of Bern, Bern, Switzerland

**Keywords:** focus group, patient reported outcome, qualitative research, solid organ transplantation, thematic analysis

## Abstract

Solid organ transplantation has achieved remarkable improvements in graft and patient survival, making traditional biomedical outcomes alone insufficient to fully define transplant success. Thus, attention is growing directed toward patient-reported outcomes, quality of life, psychological wellbeing, treatment burden, social reintegration, and the broader lived experience of transplant recipients. These dimensions are often subjective and context-dependent making them difficult to capture through quantitative methods alone. Qualitative research offers a complementary approach by exploring how patients, caregivers, donor families, and healthcare professionals perceive and give meaning to transplantation. In transplant medicine, qualitative methods may represent a relevant resource. Despite its potential, qualitative research remains underrepresented in leading transplantation journals and is often limited by misconceptions regarding rigor, lack of methodological training, and lacking editorial know-how. Integrating qualitative, quantitative, and mixed-methods approaches may help generate more comprehensive evidence. A new research agenda in transplantation should therefore move beyond graft survival to include lived experience as a central component of patient-centered care.

## Introduction

Over the past decades, the field of solid organ transplantation (SOT) has witnessed remarkable progress. Advances in peri- and post-operative care have led to substantial improvements in both graft and patient survival, with the 5-year patient survival rate exceeding 90% for the majority of organs [1–4]. Consequently, outcomes that were once the primary focus of clinical research, namely, short- and long-term survival, while still fundamental, are no longer sufficient to fully define transplant success. Fowler [5] reinforces this view by highlighting a *false narrative* in kidney transplantation, where graft survival is seen as the sole definition of success, while critical dimensions of adaptation to life after a transplant are overlooked. Thus, interest has been shifting toward a broader and more patient-centered definition of success [6]. Patient-reported outcomes (PROs), quality of life, psychological wellbeing, treatment burden, and social reintegration are becoming central endpoints [7]. These dimensions reflect what truly matters to patients living with a transplant, extending beyond traditional biomedical metrics [8]. However, these outcomes pose a significant methodological challenge. Many of these constructs are subjective, context-dependent, and deeply influenced by individual experiences. While quantitative research remains essential, it may not always be sufficient to fully explore such nuanced domains. In fact, at the core of PRO development, prior to measure standardization, lies the identification of patient priorities via rigorous qualitative research [9]. In this context, qualitative research offers a powerful and complementary approach.

## What is qualitative research, and why does it matter?

Qualitative research focuses on understanding human experiences, perceptions, and behaviors within their natural context, answering “why” and “how” questions. It can be defined as an interpretive approach to data collection and analysis, concerned with the meanings people attach to their experiences of the social world [10]. In practice, qualitative research serves different purposes depending on the study goals and the researchers’ perspectives [11, 12]. In some instances, it acts as a supportive tool used to refine clinical instruments or add narrative depth to quantitative findings; in these cases, the focus is often on reducing researcher bias, standardizing data collection, and using pre-established categories of analysis to ensure consistency. In other instances, qualitative research is undertaken as an independent process that treats the participant as the expert of their own experience. Here, the goal is not to standardize questions or responses, but to uncover the meanings and constructions that shape health and illness, and acknowledging that subjectivity is not a “bias” to be eliminated, but the essence of the data being studied.

In the field of SOT, where medical interventions can be life-transforming, qualitative research can provide insights into areas that are otherwise difficult to quantify. These include patients lived experiences before and after transplantation, their expectations and fears, adherence behaviors, perceptions of immunosuppressive therapy, and the psychosocial impact of long-term follow-up. It can also capture caregivers’ needs and responsibilities, which may otherwise remain unseen, as well as healthcare providers’ perspectives.

## Potential applications in transplant medicine

The potential applications of qualitative research in transplantation are broad and still underexplored [13]. There is a growing interest in PRO measures as valuable outcome, beyond survival, aiming to quantify quality of life and symptoms burden [6]. The field of kidney transplantation, for instance, is currently at the forefront of PRO initiatives [14]. While PRO measures attempt to quantify these dimensions, they originate from inherently subjective and experiential domains. Qualitative research can help identify what patients themselves consider important, thereby refining existing tools or contributing to guide the design of interventions that are better aligned with patients’ needs [15]. Importantly, qualitative methods can help uncover dimensions that may not be anticipated *a priori* and therefore may be missed by structured quantitative instruments. Similarly, qualitative approaches are critical in other domains beyond PRO. For instance, the theme of adherence to immunosuppressive medications remains a major challenge in transplantation; while quantitative studies can measure adherence rates, qualitative approaches can explore the underlying reasons, beliefs, and barriers that drive patient behavior, yielding insights that are far more relevant for clinicians aiming to improve adherence [16]. The utility of qualitative methods extends into interpersonal dynamics of care, particularly to inform shared decision-making. Decisions around transplant eligibility, listing, and post-transplant management are complex and often involve value-laden considerations. Understanding how patients and clinicians perceive risks, benefits, and trade-offs can improve communication and support more informed and individualized decisions.

Beyond clinical interactions, qualitative research is essential for capturing the profound existential and emotional dimensions of transplantation. For the recipient, this may include the complex reconstruction of identity [17], or the navigation of emotions such as survivor’s guilt [18]. Similarly, the impact of transplantation extends beyond the patient to include, for example, donor families and their experiences of grief associated with deceased donation [19]. Even healthcare providers are not immune to these complexities; as evidenced by studies on the emotional toll faced by transplantation coordinators [20].

Finally, qualitative methods can contribute to health system and policy research. They can explore disparities in access to transplantation, cultural and social determinants of care, and patient experiences across different healthcare settings [21].

## Challenges and misconceptions

Despite its potential, qualitative research remains underutilized in transplantation medicine. Several barriers contribute to this gap and are not unique to transplantation. Calls to overcome the marginalization of qualitative research in medicine extend back several decades [22, 23], yet subsequent analyses have continued to document its limited representation in medical journals that remain relevant today [24]. A first major barrier relates to perception. Qualitative research is sometimes viewed as less rigorous or as “second-tier” research. This misconception often stems from a lack of familiarity with its methodological foundations. High-quality qualitative research follows well-established frameworks, including sampling strategies, systematic data collection, rigorous analytical processes, and transparency in reporting, however there is no single set of quality criteria applicable across all qualitative approaches [25]. Quality should be assessed in relation to the philosophical assumptions, and methodological approach used in each specific study. Concepts such as credibility, dependability, transferability, and confirmability are influential within some qualitative traditions [26]. Broadly, these concepts concern whether the findings are well supported by the data, whether the research process is coherent and transparently described, and whether the findings may be relevant in contexts beyond the specific study. Other traditions place greater emphasis on reflexivity, interpretive depth, and the role of the researcher [27, 28]. The key principle is therefore to achieve methodological coherence, that is, remaining consistent within the approach and the claims being made [25].

This perception gap is supported by empirical evidence. In a qualitative study involving medical trainees and physicians, participants consistently rated quantitative projects as more “scientific” than qualitative ones and often perceived qualitative data as more biased and less objective. Notably, acceptance of qualitative research was strongly associated with prior exposure to its methods, while level of training had no impact [27]. In fact, most clinicians and clinical researchers are primarily trained in quantitative methodologies and have limited exposure to qualitative approaches. A recent published study examining how medical faculty without qualitative research experience perceived its challenges and potential in health research found that despite recognizing its value, academics struggled to engage in qualitative research due to limited training, experience, and institutional support. Time constraints and lack of recognition further hindered its adoption. Also, the lack of targeted mentoring and hands-on training was pointed as problematic [29]. As a result, they may feel uncomfortable designing, conducting, or interpreting qualitative studies.

This also translates into a major challenge for the publication of such studies in journals primarily targeted to a transplant clinical or basic science audience, which largely operate within a quantitative framework. A simple PubMed search, using leading journals in SOT combined with keywords related to qualitative research (such as “qualitative,” “interviews,” “thematic analysis,” and “focus groups”) and restricted to the past 5 years, shows that less than 2% of published articles fall within this domain (Figure 1). This underrepresentation is not solely a deliberate editorial choice related to target audience or journal metrics (such as impact factor), but also reflects the limited availability of editors and reviewers with expertise in qualitative methodologies. As a result, the quality and relevance of submitted work may be difficult to assess, leading to inconsistent evaluation standards and lower acceptance rates. In turn, this discourages qualitative researchers from submitting to these journals, further widening the gap.

**FIGURE 1:**
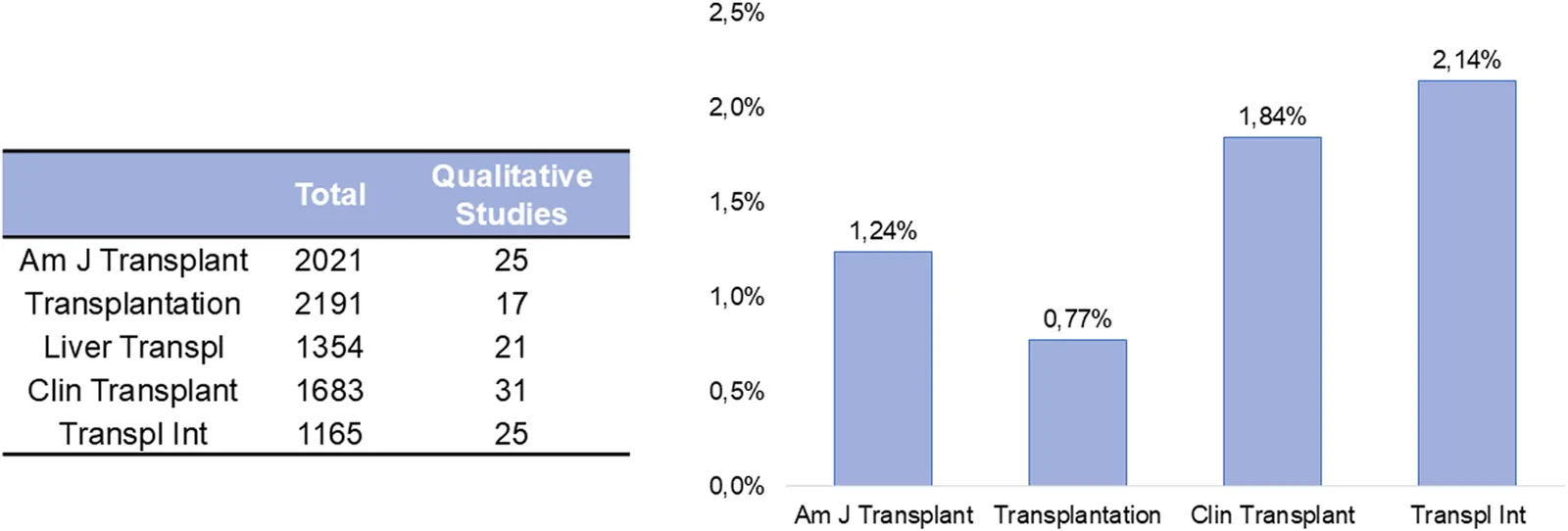
Proportion of qualitative research articles in leading SOT journals over the past 5 years.

A common example is the criticism that qualitative samples are “too small”. Most qualitative studies, however, do not seek statistical representativeness or determine sample size through statistical power. In qualitative research, sampling is most often purposive and oriented toward generating sufficiently rich and relevant data to address the research question [30]. Concepts such as saturation, are appropriate within some traditions (e.g., grounded theory) and should not be imposed as universal criteria [30]. Other approaches draw on concepts such as information power which considers whether the participants and data provide sufficient relevant information to answer the research question, rather than judging sample adequacy by the number of participants alone. These principles should therefore be applied in accordance with the specific methodological approach [31].

Similarly, qualitative research should not be reduced to conducting interviews and summarizing responses. Different forms of data collection can be employed, including interviews, focus groups, observations, documents, and other textual or visual materials, depending on research question and context [32, 33]. The analysis of data also requires enough methodological expertise or expert guidance to ensure that data generation, analysis, and interpretation are coherent within the chosen approach. Even familiar methods such as interviews require skills in listening, questioning, probing, rapport building, and reflexive awareness [33].

These systemic issues are often rooted in a confusion of research intentions; reviewers frequently apply quantitative principles to qualitative work, demanding outcomes such as generalizability or objectivity, which are fundamentally mismatched with the specific approach being employed.

Clarke et al. [34] have recently highlighted these issues, reporting on qualitative researchers’ experiences of methodologically incongruent feedback during the peer review process. The contributors to this research highlighted how reviewers and editors often lack appropriate methodological expertise. When attempts to clarify these issues fail, researchers may feel compelled to compromise methodological rigor to achieve publication, an issue particularly pronounced among early-career academics within a “publish or perish” environment.

Reporting guidelines and methodological resources, such as the Consolidated Criteria for Reporting Qualitative Research (COREQ) checklist [35], can support greater transparency and facilitate appraisal of qualitative research. More recently, reporting guidelines have increasingly become specific to particular methodologies or study designs, recognizing important differences even within qualitative research. However, these tools should be selected and interpreted in relation to the methodology employed rather than treated as universal quality checklists; appropriate reporting does not substitute for methodological coherence [25]. We provide key resources for readers seeking further guidance in Table 1.

**TABLE 1:** Key methodological resources.

References	Type
Kuper, A., Reeves, S., and Levinson, W. (2008). An introduction to reading and appraising qualitative research. BMJ, 337 [32]	General methods
Pope, C., and Mays, N. (Eds.). (2019). Qualitative research in healthcare. 4th ed. Wiley-Blackwell [33]	General methods
Braun, V., and Clarke, V. (2013). Successful qualitative research: A practical guide for beginners [36]	General methods
Malterud, K., Siersma, V. D., and Guassora, A. D. (2016). Sample size in qualitative interview studies: guided by information power. Qualitative health research, 26(13), 1753–1760 [30]	General methods
Tong A, Sainsbury P, Craig J. Consolidated criteria for reporting qualitative research (COREQ): a 32-item checklist for interviews and focus groups. Int J Qual HealthCare. 2007; 19(6):349–357 [35]	Reporting guidance
O'Brien BC, Harris IB, Beckman TJ, Reed DA, Cook DA. Standards for reporting qualitative research: a synthesis of recommendations. Acad Med. 2014; 89(9):1245–1251 [37]	Reporting guidance
Braun, V., and Clarke, V. (2025). Reporting guidelines for qualitative research: A values-based approach. Qualitative Research in Psychology, 22(2), 399–438 [25]	Reporting guidance
Tong, A., Chapman, J. R., Israni, A., Gordon, E. J., and Craig, J. C. (2013). Qualitative research in organ transplantation: recent contributions to clinical care and policy. *American Journal of Transplantation*, *13*(6), 1390–1399 [38]	Transplantation
Jamieson, N. J., Hanson, C. S., Josephson, M. A., Gordon, E. J., Craig, J. C., Halleck, F., and Tong, A. (2016). Motivations, challenges, and attitudes to self-management in kidney transplant recipients: a systematic review of qualitative studies. American Journal of Kidney Diseases, 67(3), 461–478 [39]	Transplantation
Tong, A., Morton, R. L., and Webster, A. C. (2016). How qualitative research informs clinical and policy decision making in transplantation: a review. Transplantation, 100(9), 1997–2005 [40]	Transplantation

## Integrating qualitative and quantitative approaches

Rather than viewing qualitative and quantitative research as competing paradigms, qualitative research can stand independently or be integrated with quantitative approaches when this is appropriate to the research question. Mixed-methods approaches, which integrate both types of data, are particularly promising in transplantation medicine [41]. Quantitative data can provide generalizable evidence on outcomes, while qualitative data can offer depth and contextual understanding, helping to interpret and apply those findings in real-world settings. A good example of this integration is found in clinical trial research. Traditionally, qualitative components in trials have been treated as subordinate “add-ons” or “nested” sub-studies [42]. However, researchers are being encouraged to plan comprehensive studies rather than just trials [43]. In this mixed methods model, the qualitative component is an equal partner that can optimize recruitment, refine informed consent, and explain unexpected outcomes, thus transforming the trial’s contributions in ways that single designs alone cannot deliver.

To fully realize the potential of qualitative research, several steps are needed. Training programs should incorporate qualitative methods into clinical research curricula, equipping clinicians with the skills needed to design and interpret such studies. Interdisciplinary collaboration with social scientists and qualitative methodologists should be encouraged. Journals should also strive to include editors and reviewers with expertise in qualitative research and develop clearer guidelines for its evaluation.

In conclusion, as transplantation outcomes improve, redefining success beyond survival to include patient experience and quality of life highlights the critical role of qualitative research in advancing truly patient-centered care.

## Data Availability

The original contributions presented in the study are included in the article/supplementary material, further inquiries can be directed to the corresponding author.
